# *In vitro* assembly of Ebola virus nucleocapsid-like complex expressed in *E. coli*

**DOI:** 10.1007/s13238-016-0314-1

**Published:** 2016-09-20

**Authors:** Ruchao Peng, Tengfei Zhu, Babayemi Olawale Oladejo, Abednego Moki Musyoki, Yingzi Cui, Yi Shi, Peiyi Wang, George Fu Gao

**Affiliations:** 1 CAS Key Laboratory of Pathogenic Microbiology and Immunology, Institute of Microbiology, Chinese Academy of Sciences, Beijing, 100101 China; 2 University of Chinese Academy of Sciences, Beijing, 101408 China; 3 Research Network of Immunity and Health (RNIH), Beijing Institutes of Life Science, Chinese Academy of Sciences, Beijing, 100101 China; 4 Faculty of Biological Sciences, University of Leeds, Leeds, LS2 9JT UK; 5 National Institute for Viral Disease Control and Prevention, Chinese Center for Disease Control and Prevention (China CDC), Beijing, 102206 China

## Abstract

Ebola virus (EBOV) harbors an RNA genome encapsidated by nucleoprotein (NP) along with other viral proteins to form a nucleocapsid complex. Previous Cryo-eletron tomography and biochemical studies have shown the helical structure of EBOV nucleocapsid at nanometer resolution and the first 450 amino-acid of NP (NPΔ451–739) alone is capable of forming a helical nucleocapsid-like complex (NLC). However, the structural basis for NP-NP interaction and the dynamic procedure of the nucleocapsid assembly is yet poorly understood. In this work, we, by using an *E. coli* expression system, captured a series of images of NPΔ451–739 conformers at different stages of NLC assembly by negative-stain electron microscopy, which allowed us to picture the dynamic procedure of EBOV nucleocapsid assembly. Along with further biochemical studies, we showed the assembly of NLC is salt-sensitive, and also established an indispensible role of RNA in this process. We propose the diverse modes of NLC elongation might be the key determinants shaping the plasticity of EBOV virions. Our findings provide a new model for characterizing the self-oligomerization of viral nucleoproteins and studying the dynamic assembly process of viral nucleocapsid *in vitro*.

## Introduction

Ebola virus (EBOV) is the representative species of *Filoviridae* family, including another two members, Marburg virus (MARV) and Lloviu virus (LLOV), which causes severe hemorrhagic fever with extremely high morbidity and mortality in humans and non-human primates (Kuhn et al., 2010). Typically, EBOV is circulating discretely within some regions in Central and Southern Africa. However, at an unprecedented scale, the emergence in Western Africa in 2014 raged with an extreme severity spreading to many regions in Africa and some cases of occasional infection (most of them were Africa-importing cases) were also reported outside Africa, raising the great potential of a worldwide prevalence (http://apps.who.int/gho/data/node.ebola-sitrep) (Gao and Feng, 2014). Though the outbreak is over with the great efforts made by many people all over the world fighting at the frontline, this event revealed our great shortage of effective vaccines and therapeutics, or a detailed understanding of its biology. EBOV yet remains a great threat to the public health worldwide.

EBOV belongs to the *Mononegavirales* order, *Filoviridae* family, characterized as a non-segmented negative-strand RNA virus (Kuhn et al., 2010). The genomic RNA of these viruses exists as ribonucleoprotein (RNP) complex, or nucleocapsid, with nucleoprotein (NP) binding along with other viral proteins (Ruigrok et al., 2011; Zhou et al., 2013). Both the transcription and replication processes are accomplished within the RNP complex which directly serves as the template for viral RNA dependent RNA polymerase (RdRp) (Zhou et al., 2013). The nucleocapsid complex of EBOV is composed of the genomic RNA, NP, virion-associated protein (VP) 30, VP24, VP35 and L protein (the viral RdRp) (Huang et al., 2002; Noda et al., 2006). Upon infection, the virion would be engulfed into the endosome and then the viral envelop would fuse with the endosome membrane to release the nucleocapsid into the cytoplasm, initiating the viral gene expression and genome replication (Alvarez et al., 2002; Miller et al., 2012; Nanbo et al., 2010; Hunt et al., 2011; White and Schornberg, 2012; Wang et al., 2015; Gong et al., 2016). During the whole life cycle of EBOV, the nucleocapsid complex serves as the basic functional unit of its genome and is therefore an important target for anti-viral intervention.

The EBOV nucleocapsid complex was shown to form a helical structure by previous Cryo-electron tomography (Cryo-ET) studies at nanometer resolution (Noda et al., 2005; Bharat et al., 2012; Beniac et al., 2012). Biochemical and biophysical studies on NP, the main component of viral nucleocapsid, defined the boundary between N-terminal and C-terminal domains and mapped the functional region responsible for NP-NP interaction to be within the first 450 amino acids (Watanabe et al., 2006; Noda et al., 2010). Though the full-length EBOV NP comprises 739 amino acids, recombinant NPΔ451–739 protein expressed in mammalian cells grabs non-specific cellular RNA and self-assembles into helical structure, a nucleocapsid-like complex (NLC), with similar morphology of the authentic viral nucleocapsid (Noda et al., 2010).

Due to the high propensity of self-assembly and heterogeneity, structural investigations on NP is extremely challenging. Recently, the crystal structures of the truncated NP C-terminal domain (CTD) and N-terminal domain (NTD) were reported (Leung et al., 2015; Dong et al., 2015; Kirchdoerfer et al., 2015; Dziubanska et al., 2014), providing insights into the structural fold of the NP protein. However, these truncated proteins were incapable of assembling into NLC or encapsidating RNA. The detailed molecular mechanism for the dynamic process of nucleocapsid assembly yet remains enigmatic.

In this regard, our current study showed that the NPΔ451–739 protein expressed in *E. coli* indeed assembled into an NLC. We further observed the dynamic process of EBOV NLC assembly *in vitro* by electron microscopy (EM) and investigated multiple factors that affect the assembly process using this *E. coli* expression system. Our approach would further expand our knowledge regarding the nucleocapsid formation of all non-segmented negative-strand RNA viruses.

## Results

### *In vitro* assembly of the ring-like EBOV NLC particles expressed in *E. coli*

**Figure 1:**
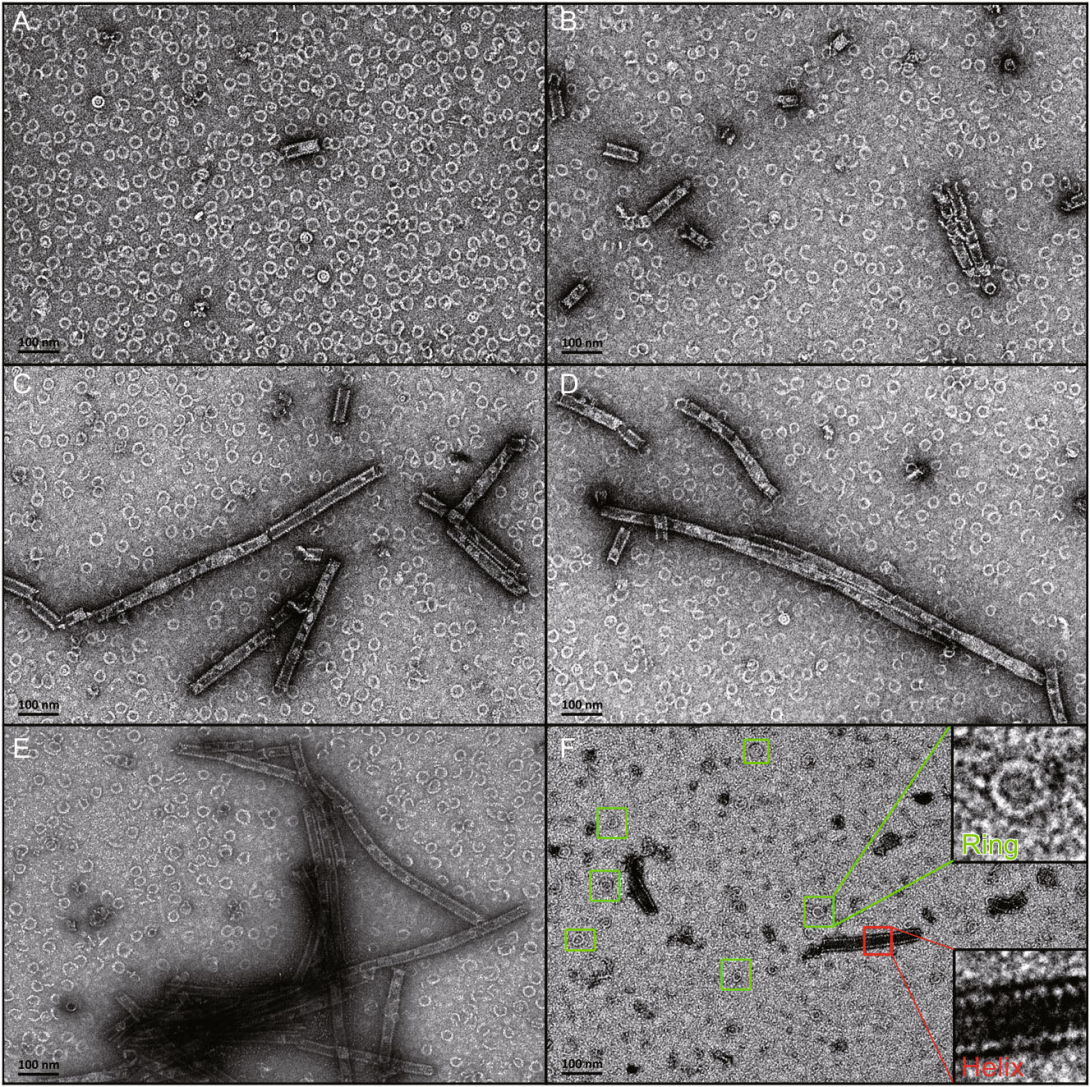
***In vitro*** **NLC assembly time lapse**. (A) The freshly purified EBOV NPΔ451–739 protein was immol/Lediately applied for negative stain EM observation. (B–E) The EBOV NPΔ451–739 protein preparation at a concentration of ~1 mg/mL was kept at 4°C for 2 days and samples were taken every 6 h and observed by negative-stain TEM. A, ring formation; B, short helix nucleation; C, helix elongation; D, long helix assembly; E, long helix aggregation. (F) Mammol/Lalian cell (293T cell) expressed EBOV NPΔ451–739 protein purified following the same protocol as described above for *E. coli* expressed samples. The cells were homogenized by sonication. The ring particles and helices are labeled by green and red rectangles, respectively

Previous work by several groups has demonstrated that the N-terminal domain of EBOV NP is responsible for NP-NP interaction and the first 450 amino acids is sufficient for a helical NLC formation (Watanabe et al., 2006; Noda et al., 2010). Therefore, we adopted a similar strategy to express the recombinant NPΔ451–739 protein in *Escherichia coli* (*E. coli*) system and examine the morphology of the protein particles by negative-stain EM. Surprisingly, we observed a large proportion of ring-like particles besides the helical NLC (Fig. 1A), which were approximately 30 nm in diameter and have never been reported in any previous studies on EBOV NP. In addition, both the percentage and length of helical NLC particles increased if the samples were kept at 4°C for a certain period (Fig. 1A–E).

In the beginning, the freshly prepared NPΔ451–739 protein sample mainly formed ring structure with rather few helical structured particles. Looking at the particles in detail, we found they were varied in size and shape, indicating the different intermediates in the process of NP oligomerization (Fig. 1A). As the protein preparations were kept at 4°C for a certain period of time and samples were taken regularly to make a time lapse, an obvious increment in both the length and percentage of the NLC particles was observed (Fig. 1A–E). Meanwhile, we also noticed many particles with irregular morphology, indicating the intermediates in the course of helix formation. Given long enough time, the helical NLC particles would interfere with each other and tend to aggregate (Fig. 1E), which might be due to the hydrophobic nature of the NPΔ451–739 protein. These observations above indicated that the NLC was built up with the ring-like particles as the building block and this assembly process could be revealed *in vitro* with protein expressed in *E. coli*.

### Reversibility of NLC assembly

Despite that many groups have made great efforts to characterize the EBOV nucleocapsid complex both functionally and structurally, no one has ever described the 30 nm ring-like particles of EBOV NP. Kawaoka’s group pioneered in this field (Noda et al., 2006; Noda et al., 2005; Watanabe et al., 2006; Noda et al., 2010). In the previous biochemical and biophysical studies, they first discovered that recombinant expression of the first 450 amino acids of EBOV NP in mammalian cells could generate helical structured NLC particles with non-specific cellular RNA binding (Watanabe et al., 2006; Noda et al., 2010). Our observation of ring-like particles was made by expressing the protein in *E. coli* system.

**Figure 2:**
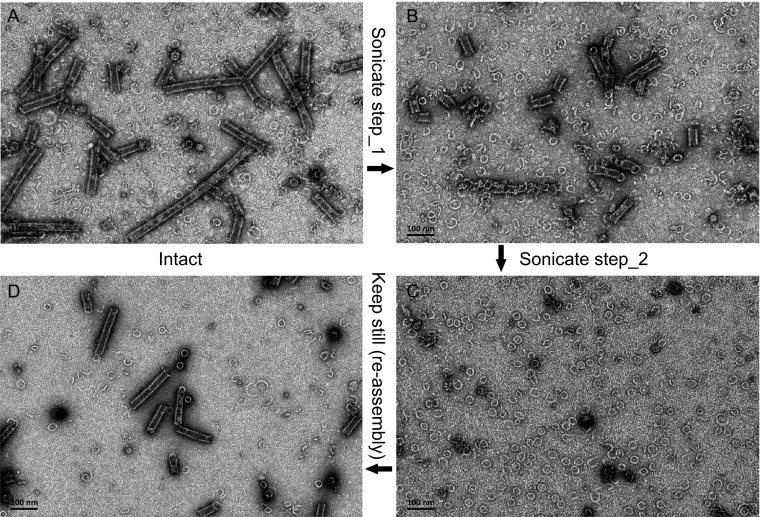
**Reversible NLC assembly** ***in vitro***. The pre-assembled NLC sample (A) was treated by sonication on ice (sonicate for 1 s with 2 s interval, 200 W, 10 (B) /20 (C) cycles) to disrupt the helical structure. And then the sonicated sample was kept at 4°C for 24 h (D)

To test the reversibility of this process, we treated the pre-assembled helical NLC preparations with sonication on ice and visualized the resulting particles by negative-stain EM. Consequently, the helical NLC particles largely dissociated into ring-like particles (Fig. 2B and 2C). Accordingly, the disrupted ring particles would reversibly re-assemble into helical NLC particles if kept at 4°C for a certain period of time (Fig. 2D). On the other hand, we prepared the NPΔ451–739 protein expressed in 293T cells following the same purification protocol and examined the morphology of the protein particles by negative-stain EM as well. The ring-like particles were also observed in the 293T cell expressed NPΔ451–739 protein preparations along with the helical NLC particles (Fig. 1F).

Our data showed the dynamic process of NLC assembly was reversible *in vitro*, providing us a novel model for investigating the dynamics of the NLC assembly of non-segmented negative-strand RNA viruses.

### Dynamic procedure of the NLC assembly

**Figure 3:**
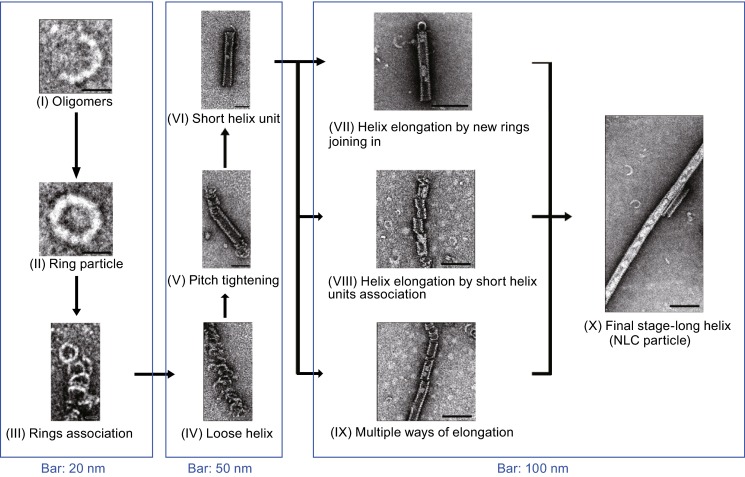
**Dynamic process of NLC assembly**. The different conformers captured by EM at different stages of NLC assembly process showing the dynamics of EBOV NLC assembly model. Each stage is given by a micrograph of representative particles

All the above observations imply a process of helical NLC particles assembly from the ring-like particles, originally from the monomeric NPΔ451–739 protein. Therefore, freshly prepared NPΔ451–739 protein was subjected to make a more detailed time-lapse EM analysis to capture the various conformers at different stages of NLC assembly. As expected, a series of intermediate NPΔ451–739 protein particles were successfully observed in the assembly process from ring formation to final NLC helical structure (Fig. 3), allowing us to picture the whole dynamic procedure by logical analysis.

Once the NPΔ451–739 protein was expressed in the cell, it tends to oligomerize and gather adjacent molecules to form ring-like particles (Fig. 3_II), which would serve as the building block for further NLC assembly. Due to the physical disruption, we also observed some incomplete ring particles (Fig. 3_I), representing the precursors for ring formation. Right after that, the ring particles would associate with each other to generate the loose helical structured particles characterized by the variable helical pitch distance and irregular shape (Fig. 3_III-IV). This conformation is extremely flexible and would soon further condense by tightening the pitches, resulting in tight helix unit with near constant pitch distance and more regular morphology (Fig. 3_V-VI). At this stage, the helix unit is universally very short and would further serve as the nuclear scaffold for elongation. As to helix elongation, there are multiple ways to accomplish the process. Both ends of the helix unit could be elongated by new rings joining in (Fig. 3_VII). Several adjacent short helix units could also associate together to generate a longer helix by aligning the axis and tightening the gaps (Fig. 3_VIII). Alternatively, both the two elongation processes could happen simultaneously, i.e. ring-like particles and short helix units could be recruited and aligned together directly (Fig. 3_IX). Eventually, the long helical NLC particles formed (Fig. 3_X).

With the accumulation of long helical NLC particles, they tend to interfere with each other and aggregate, which might result from the interactions of exposing hydrophobic interfaces of adjacent NLC particles (Fig. 1E).

### Structural plasticity and heterogeneity of the helical EBOV NLC particles

**Figure 4:**
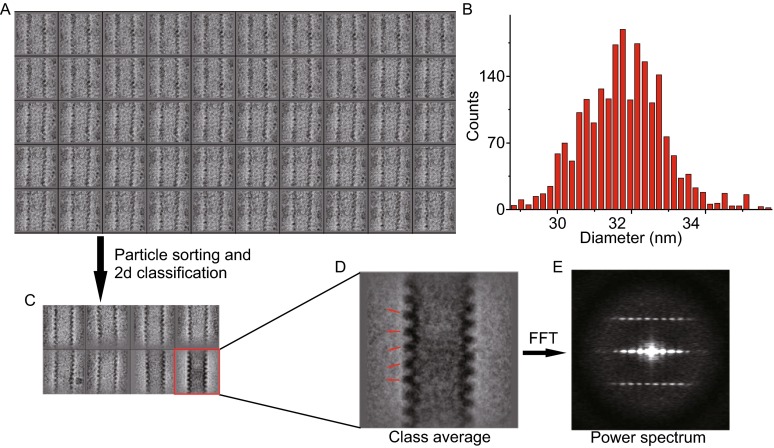
**Preliminary image processing of helical EBOV NLC particles**. Preliminary 2D classification and average of helical NLC particles. (A) Selected segmented short EBOV NLC helices raw images. (B) The helix diameter distribution histogram of NLC particles. (C) 2D classification and average of most populated helix image set. (D) Zoom in of the best 2D class average image. (E) The power spectrum of the best class average

We then did some preliminary image processing and analysis for helical EBOV NLC particle micrographs. By direct visual inspection, we noticed that the helical NLC particles was not straight but with certain extent of twist (Fig. 1C–E, Fig. 4A), remarkably different from the canonical rigid helix, *e,g.* tobacco mosaic virus (TMV) virion and microtubes. This is reminiscent of the plastic authentic EBOV virion, indicating the NLC particles could to some extent reveal the physiological properties of EBOV nucleocapsid.

We then measured the diameter of NLC helices and it showed a rather broad range distribution, ranging from 28 to 36 nm (Fig. 4B), further demonstrating the structural heterogeneity of NLC particles. After sorting the NLC helix particles by diameter, the most populated set, with a diameter of 30–32 nm, was picked out and subjected for 2D classification and averaging. The best class average with the highest signal to noise ratio and contrast was selected and some more detailed structural features could be observed. We noticed that the helix was not regularly periodic but with obvious pleomorphism (Fig. 4C and 4D), i.e. the helical pitch distance was not constant and the direction of each turn was not uniform either, which was further evidenced by its poor power spectrum pattern without recognizable layer lines (Fig. 4E).

This flexibility of NLC particles to some extent reveals the physiological properties of EBOV nucleocapsid, even the virion, and makes the structural studies on its assembly mechanism extremely challenging.

### The assembly of NLC particles is salt-sensitive

Though the assembly of NLC particles basically relies on the self-interactions of NPΔ451–739 protein, there are several biochemical factors that affect the assembly process and may change the morphology of NLC particles. Previous work by Noda et al. (2010) showed that the morphology of NLC particles is salt-sensitive as a result of NP conformational change in response to environmental salt concentration changes (Noda et al., 2010). Here we also demonstrate a critical role of salt concentration in the assembly procedure of NLC particles.

**Figure 5:**
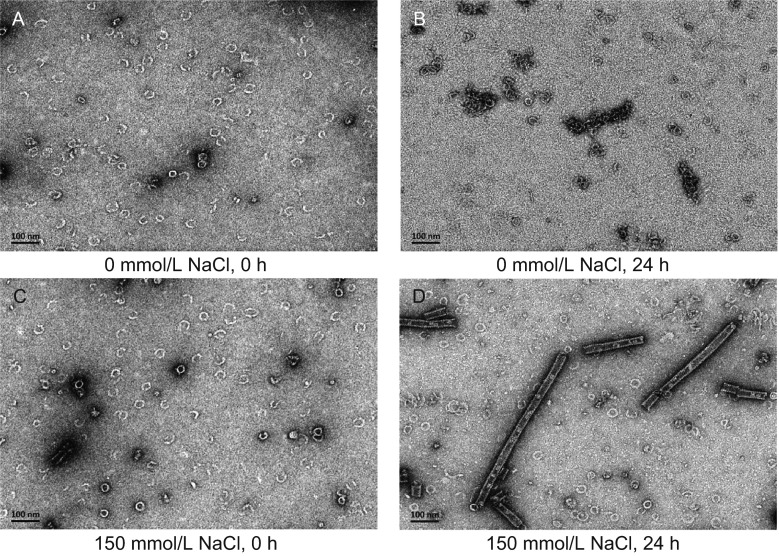
**Effect of salt concentration on the assembly of NLC particles**. The freshly purified NPΔ451–739 protein in buffer containing 20 mmol/L Tris-HCl, 150 mmol/L NaCl, pH 8.0 (C) was buffer-exchanged into salt free buffer (A). And then the two samples were kept still at 4°C for 2 days to compare the NLC assembly process with different salt conditions. Take samples every 12 h and observe by negative-stain EM. (B) and (D) show the result at 24 h for assembly

We purified the NPΔ451–739 protein in different buffer conditions with either physiological salt concentration or salt-free, respectively, and then examined the morphology of the protein particles by negative-stain EM. The freshly prepared protein samples at both conditions showed similar ring structure indistinguishable between each other (Fig. 5A and 2C), indicating the high stability of NP-NP interactions to maintain the ring structure. After being kept at 4°C for a certain period of time, the NPΔ451–739 protein particles in buffer with physiological salt concentration assembled into helical NLC particles as described above (Fig. 5D). However, the sample in salt-free buffer was unable to form NLC particles though the ring particles did associate together to some extent without the correct orientation for helix axis alignment, forming irregular shaped aggregates (Fig. 5B). Together with Noda’s previous studies, our data demonstrate that salt concentration plays an important role in both the assembly process of the NLC particle and maintaining its structure.

### RNA is essential for NLC assembly but not for maintaining its helical structure

Self-assembly and encapsidating RNA are two main functions of viral NPs. In Noda’s previous work, the recombinant expressed NP protein in mammalian cells, both full length and constructs with C-terminal truncation (NPΔ451–739 and NPΔ601–739), harbors non-specific cellular RNA (Noda et al., 2010). Similarly, we also found the recombinant NPΔ451–739 protein expressed in *E. coli* with nucleic acid binding, indicated by an OD_260_/OD_280_ of ~1.2, and it is present throughout the whole procedure of NLC assembly. Therefore, we set out to characterize the nucleic acid encapsidated by NPΔ451–739 and investigate its role in the assembly process of NLC particles.

**Figure 6:**
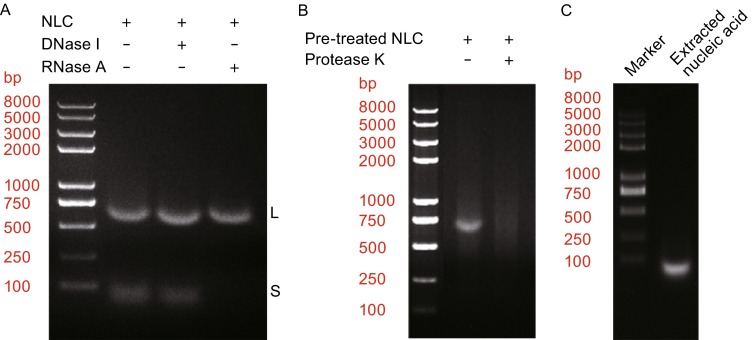
**Characterization of NPΔ451**–**739 binding nucleic acid**. (A) The NLC sample was treated with either DNase I or RNase A and analyzed by agarose gel electrophoresis. The band profile mainly includes 2 bands, L and S. (B) The NLC sample was digested with RNase A and purified by Superose 6 10/300 GL (GE Healthcare) size exclusion chromatograph to remove the remaining RNase A. The resulting product (lane 2) was digested with protease K (lane 3) and analyzed by agarose gel electrophoresis. (C) Agarose gel electrophoresis of nucleic acid extracted from EBOV NLC particles by phenol-chloroform- isopentanol method. Only the S-species could be observed

We first performed agarose gel electrophoresis directly with the NLC particle sample. Two bands were clearly observed with different molecular weight, large species (L-species) and small species (S-species), demonstrating the presence of nucleic acid in the NLC particles (Fig. 6A). To further characterize the chemical nature of the NPΔ451–739 binding nucleic acid, enzymatic digestion assay was conducted with DNase I and RNase A, respectively. Interestingly, DNase I treatment did not cause any change in the band profile, indicating both nucleic acid species are not DNA, while the RNase A treatment degraded the S-species but leaving the L-species intact, which unambiguously identified the S-species as RNA but still left the L-species a mystery (Fig. 6A).

Considering the resistance to both DNase I and RNase A of the L-species, we speculate that it might be shielded by the NPΔ451–739 protein in the inner face, making it inaccessible for both enzymes. Therefore, in order to fully characterize the L-species, we must extract it from the NLC particles. Unfortunately, we were never able to extract the L-species from NLC samples though having tried many strategies, including phenol-chloroform-isopentanol method, ammonium sulfate precipitation and many other nucleic acid extraction kits. In contrast, the S-species was easily extracted and purified (Fig. 6C). Eventually, we tried protease K digestion strategy given that once the protein shield was degraded the nucleic acid would be released and could be purified. To our surprise, the L-species band disappeared after protease K digestion (Fig. 6B). This indicated that the L-species might be some really small nucleic acid or oligo-nucleatides non-specifically bound to NPΔ451–739 protein. The low mobility rate in gel-electrophoresis might result from the bound NPΔ451–739 protein shield which was not completely disrupted in electrophoresis environment. Therefore, the S-species RNA should be the mimic of viral genomic RNA encapsidated by the nucleocapsid complex. The RNase A and DNase I digestion assay demonstrates that NPΔ451–739 protein specifically recognize RNA but not DNA and the encapsidated RNA molecule is not protected by the NLC complex from RNase A digestion.

**Figure 7:**
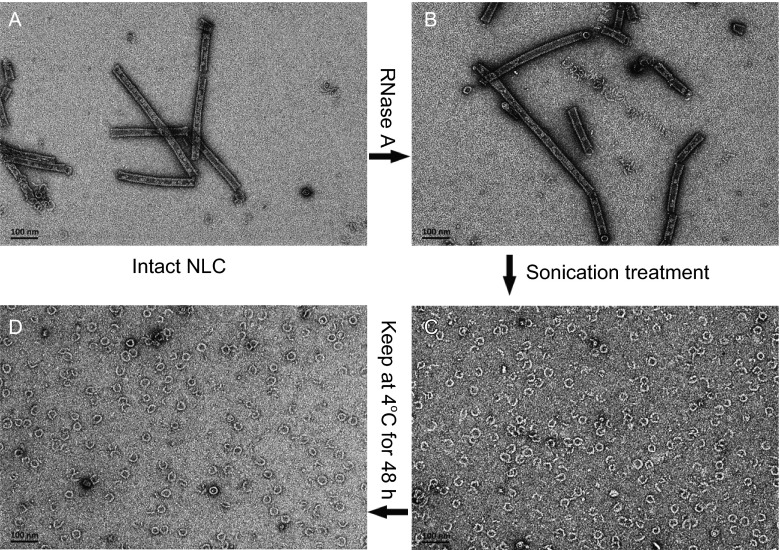
**Effect of binding nucleic acid on the NLC assembly process**. The NLC sample (A) was digested with RNase A (B), and disrupted by sonication (C), then kept still at 4°C for 2 days to test the NLC assembly process. Samples were taken and observed by negative-stain EM for every 12 h and (D) showed the result of 48 h post treatment

We then investigated the role of RNA in the NLC assembly process and maintaining its structure. Firstly, the helical NLC particles were digested with RNase A to remove the bound S-species RNA and the morphology of the intact and digested NLC particles were visualized by negative-stain EM. Both the digested and intact NLC particles showed similar helix structures without appreciable morphology changes (Fig. 7A and 7B), indicating the encapsidated RNA is not essential for stabilizing the helical structure of NLC particles. The NLC helical structure is mainly stabilized by the profound NP-NP interactions. This experiment excluded the bound RNA as an essential element for maintaining the NLC structure but still cannot define the role it played in the assembly process of NLC particles. In this regard, we disrupted the helical structure of RNase A digested NLC particles by sonication to test whether the dissociated NPΔ451–739 protein particles were able to re-assemble into helical structure in the absence of bound RNA. As it turned out, the RNase A digested sample largely remained ring structured particles after sonication treatment and was unable to re-assemble into helical structure as the undigested sample did (Figs. 7C, 7D and 2A–D), demonstrating the bound RNA an indispensible element for NLC assembly.

In conclusion, the recombinant NPΔ451–739 protein expressed in *E. coli* forms helical structure and incorporates RNA without specificity to assemble into NLC particles. The NLC helical structure cannot shield the bound RNA from enzymatic digestion. Though not essential for maintaining its helical structure, the bound RNA is an indispensible component in the assembly process of NLC particles. Our observation of *in vitro* assembly of EBOV NLC particles from ring structure to helical particles provides a novel model for studying the dynamic process of viral nucleocapsid formation.

## Discussion

Non-segmented negative-strand RNA viruses all possess an RNA genome encapsidated by nucleoprotein and other viral factors to form a nucleocapsid, which directly serves as the template for gene transcription and genome replication by the viral RdRp (Ruigrok et al., 2011; Zhou et al., 2013). Though varying in size and shape, the nucleocapsid performs similar functions to stabilize and protect the genome from host anti-viral defense and facilitate the virus replication in the host cell. It has been shown that the nucleocapsid of many members in this virus group forms helical structure and the NP plays a critical role for its assembly and provides the basic scaffold for genome accommodation (Ruigrok et al., 2011; Zhou et al., 2013; Iseni et al., 1998; Bhella et al., 2002). The nucleocapsid compositions vary between different viruses. In VSV (Vesicular Stomatitis Virus), Rabies virus, RSV (Respiratory Syncytial Virus) and Nipah virus, the nucleocapsid is mainly composed of NP, P protein (phosphoprotein) and the RdRp (Ruigrok et al., 2011; Iseni et al., 1998; Bhella et al., 2002; Yabukarski et al., 2014). In contrast, the nucleocapsid of EBOV is a much larger complex consisting of NP, VP24, VP30, VP35 and L protein (RdRp) (Huang et al., 2002; Noda et al., 2006). The composition complexity of EBOV nucleocapsid provides a vast platform for undertaking multiple functions at different stages in the virus life cycle and probably also leads to the significant plasticity of the virus morphology, which distinguishes EBOV critically from other non-segmented negative-strand RNA viruses (Beniac et al., 2012; Booth et al., 2013).

The functional region responsible for EBOV NP-NP interaction and the core helix formation has been mapped to the first 450 amino acids of NP and it is sufficient to bind RNA and support genome replication (Watanabe et al., 2006). Recombinant expression of NPΔ451–739 in mammalian cells would generate a helical NLC structure with smaller pitch distance as compared with the authentic EBOV nucleocapsid and harboring non-specific cellular RNA (Bharat et al., 2012; Watanabe et al., 2006). In our work, the recombinant NPΔ451–739 protein was expressed in *E. coli* system and showed not only helical structure but also quite a proportion of ring-like particles, roughly 30 nm in diameter, as observed by EM. Though not having been reported in any previous work on EBOV NP, similar ring-like particles were observed in several other viral NPs as well, for instance, VSV, Rabies virus and RSV (Iseni et al., 1998; Bhella et al., 2002; Albertini et al., 2006; Tawar et al., 2009; Green et al., 2006; Maclellan et al., 2007). It is noteworthy that in Leung’s and Kirchdoerfer’s previous work they also observed ring structured NP oligomers by preparing NPΔ458–739 and NPΔ451–739 protein alone respectively without RNA binding (Leung et al., 2015; Kirchdoerfer et al., 2015). However, the size of these ring particles were averagely around 40 nm in diameter, which is far larger than the diameter of NP comprised inner layer of EBOV nucleocapsid established by previous cryo-ET reconstructions (Bharat et al., 2012; Beniac et al., 2012). In contrast, the ring particles we observed in this study are in good accordance with the cryo-ET models in size, which implies that the ring particles we observed may have some functional correlations with the real viral nucleocapsid. Besides, we also observed a process of transition from ring particles to helical NLC particles *in vitro*. We then figured out that this process could be reverted automatically *in vitro*. This strategy for NPΔ451–739 protein preparation provides a novel and ideal model for studying the dynamics of EBOV NLC particles formation, which would further help to shed light on the underlying mechanism of nucleocapsid assembly of all non-segmented negative-strand RNA viruses.

Previously Noda et al. (2010) has shown that environmental salt concentration had a reversible effect on the morphology of EBOV NLC particles (Noda et al., 2010). At physiological salt concentration conditions, the NP protein assembles into helical NLC particles in the presence of RNA. Once subjected to salt free conditions, the tight helical NLC particles would be deformed into loose helix and this transition could be reverted if the salt concentration was recovered (Noda et al., 2010). These data demonstrate the role of salt concentration for stabilizing the NLC structure. We further examined the effect of salt concentration on the formation process of NLC particles. The NLC assembly process presented above was conducted in buffer containing 150 mM NaCl, which closely resembles the physiological salt concentration. As we replicate the assembly process in salt free buffer conditions, the NLC particles could not be observed despite given much longer time for assembly. Thus, we can conclude that salt concentration regulates the formation of NLC particles both in the assembly process and stabilizing its structure after assembly. These phenomena all might be related to the hydrophobicity of the NPΔ451–739 protein and there are probably more than one interface for NP-NP interactions, one horizontal interface for stabilizing neighbor NP protomers in the ring and one vertical interface for adjacent rings adherence. From the data above, we can infer the horizontal interface is more stable than the vertical one. This is probably the most crucial structural basis determining the plasticity of the NLC structure and further leading to the flexibility of EBOV virion.

Apart from the influence of salt concentration on EBOV NLC morphology, Noda’s work also established an essential role of RNA for keeping its plasticity (Noda et al., 2010). However, the role of RNA in the assembly process of NLC particles was unclear. In our studies, we showed that the *in vitro* assembly of NLC particles could only be achieved in the presence of RNA. More specifically, RNA is essential for guiding the ring particles to assemble into helical NLC. As to the ring particle formation, the sole NPΔ451–739 protein is sufficient to assemble into the ring structure, which is also supported by Leung’s and Kirchdoerfer’s observations that the RNA free NP(1–457 or 1–450) protein self-assembled into ring structured particles though larger than the ones we observed here (Leung et al., 2015). Once the helical NLC formed, removal of RNA would not cause any appreciable change in the morphology of NLC particles. These data together with Noda’s observations proved that RNA is an indispensable component for the assembly process of NLC particles but not required for stabilizing its helical structure which mainly relies on the vertical interface mediated NP-NP interactions instead.

Finally, as self-assembly and accommodating RNA are the two main functions of viral NPs, it is of great significance to figure out the molecular mechanism of NP-RNA interactions. However, there is no EBOV NP-RNA complex structure available so far. Recently, the crystal structure of EBOV NP N-terminal core domain was reported by several groups (Leung et al., 2015; Dong et al., 2015; Kirchdoerfer et al., 2015). The core domain structure shows similar overall fold with other viral NPs, indicating a general mechanism to encapsidate the genomic RNA (Yabukarski et al., 2014; Albertini et al., 2006; Tawar et al., 2009; Green et al., 2006). However, these structures were obtained from monomeric core proteins that unable to either assemble into NLC or bind RNA. Therefore, the detailed molecular mechanism of nucleocapsid assembly still warrants further investigation.

## Materials and methods

### Protein production

The gene of the *Zaire ebolavirus* nucleoprotein (GenBank: AAG40164.1, residues 1-450) was cloned into the pET-21a expression vector within *Nde*I and *Xho*I restriction sites following the general protocol. The accuracy of the inserts was verified by sequencing. The recombinant plasmid of EBOV NPΔ451–739 was transformed into *Escherichia coli* strain BL21(DE3) and overexpressed as a 6×His-tag fused protein at the C-terminus. The bacteria were cultured at 37°C in 2 L LB media containing 100 μg/mL ampicillin. Once OD_600_ reached 0.6, 1 mmol/L isopropyl-β-D-1-thiogalactopyranoside (IPTG) was added to induce the protein expression and the culture was further incubated for an additional 8 h. The cell was harvested by centrifugation at 8,000 ×g for 30 min at 4°C and resuspended in 100 mL lysis buffer (20 mmol/L Tris-HCl, 150 mmol/L NaCl, pH 8.0) and homogenized by sonication. The lysate was centrifuged at 20,000 ×g for 30 min at 4°C to remove cell debris. The supernatant was then loaded three times onto a Ni Sepharose^TM^ (GE Healthcare) column pre-equilibrated with lysis buffer. Resin was washed with 100 mL wash buffer (20 mmol/L Tris-HCl, 150 mmol/L NaCl, 50 mmol/L imidazole, pH 8.0) and eluted with 30 mL elution buffer (20 mmol/L Tris-HCl, 150 mmol/L NaCl, 300 mmol/L imidazole, pH 8.0). The protein was further purified on a Superose^TM^ 6 10/300 GL (GE Healthcare) column equilibrated with the buffer containing 20 mmol/L Tris-HCl, pH 8.0 with 150 mmol/L NaCl or without where specified. SDS-PAGE analysis revealed over 95% purity of the final purified recombinant protein.

For protein expression in mammol/Lalian cells (The mammol/Lalian cells used in this study refer to 293T cells, obtained from cell resource center of Shanghai Institutes for Biological Sciences, Chinese Academy of Sciences), the coding sequence of EBOV NPΔ451–739 was cloned into pCAGGS vector with a 5′-GCCACC Kozak sequence and 3′-6×His-tag coding sequence using *EcoR*I and *Nhe*I restriction sites. The 293T cells were cultured with Dulbecco’s modified Eagle’s medium (DMEM, Gibco) supplemented with 10% fetal bovine serum in 100 mmol/L Petri dishes at 37°C in the presence of 5% CO_2_ to a confluence of ~90% and transfected with 10 μg recombinant plasmid per dish. After 3 days post transfection, the cells were harvested and washed with 1× Phosphate Buffered Saline once, followed by resuspension in lysis buffer (20 mmol/L Tris-HCl, 150 mmol/L NaCl, pH 8.0) and purification following the same protocol described above for purifying protein expressed in *E. coli*.

### Negative staining and electron microscopy.

The EBOV NPΔ451–739 protein preparation was pre-treated with all kinds of conditions as required and diluted to 0.1 mg/mL in 0 or 150 mmol/L NaCl buffer. Then 5 μL sample was applied to 400 mesh copper grids coated with continuous carbon film, which had been plasma cleaned by glow charge, and negative-stained with 1% uranyl acetate. After air drying, the sample was observed on a JEOL-1400 EX electron microscope equipped with a Gatan Orius 832 CCD camera, operated at an acceleration voltage of 120 kV. For helical NLC particle data acquisition, the image was recorded on a magnification at a calibrated pixel size of 1.97 Å and defocus range of 2–6 μm.

### Image processing

All the micrographs were fully CTF-corrected in whole image with CTFFIND3 (Mindell and Grigorieff, 2003). The NLC helix particles were boxed with e2boxer.py in EMAN2 (Ludtke et al., 1999) package. Then the particles were sorted by diameter using IHRSR (Egelman, 2000) and reference free 2D classification was performed with e2refine2d.py in EMAN2 (Ludtke et al., 1999) package. The helix diameter and power spectrum was calculated with SPIDER (Shaikh et al., 2008). The histogram was generated by Origin 8.0.

### Enzymatic digestion assay

The pre-assembled EBOV NLC preparations were supplemented with DNase I or RNase A at a working concentration of 1 unit per 20 μL reaction system. The mixture was incubated at 4°C for 12 h before checking by running 1.5% agarose gel electrophoresis. The nucleic acid bands were visualized with ethidium bromide staining.
